# Endurance Exercise Increases Intestinal Uptake of the Peanut Allergen Ara h 6 after Peanut Consumption in Humans

**DOI:** 10.3390/nu9010084

**Published:** 2017-01-21

**Authors:** Lonneke M. JanssenDuijghuijsen, Klaske van Norren, Sander Grefte, Stef J. Koppelman, Kaatje Lenaerts, Jaap Keijer, Renger F. Witkamp, Harry J. Wichers

**Affiliations:** 1 Wageningen Food and Biobased Research, Wageningen University and Research, P.O. Box 17, 6700 AA Wageningen, The Netherlands; lonneke.janssen@wur.nl; 2 Nutrition and Pharmacology, Wageningen University and Research, P.O. Box 17, 6700 AA Wageningen, The Netherlands; klaske.vannorren@wur.nl (K.v.N.); renger.witkamp@wur.nl (R.F.W.); 3 Human and Animal Physiology, Wageningen University and Research, P.O. Box 17, 6700 AA Wageningen, The Netherlands; sander.grefte@wur.nl (S.G.); jaap.keijer@wur.nl (J.K.); 4 Nutricia Research, P.O. Box 80141, 3508 TC Utrecht, The Netherlands; 5 Food Allergy Research and Resource Program, Food Science and Technology, University of Nebraska-Lincoln, Rm 279 Food Innovation Center, P.O. Box 886207, Lincoln, NE 68588-6207, USA; stefkoppelman@zonnet.nl; 6 Maastricht University Medical Centre, Department of Surgery, NUTRIM School of Nutrition and Translational Research in Metabolism, P.O. Box 616, 6200 MD Maastricht, The Netherlands; kaatje.lenaerts@maastrichtuniversity.nl

**Keywords:** intestinal permeability, Ara h 6, allergen, endurance exercise, food-dependent exercise-induced anaphylaxis

## Abstract

Controlled studies on the effect of exercise on intestinal uptake of protein are scarce and underlying mechanisms largely unclear. We studied the uptake of the major allergen Ara h 6 following peanut consumption in an exercise model and compared this with changes in markers of intestinal permeability and integrity. Ten overnight-fasted healthy non-allergic men (*n* = 4) and women (*n* = 6) (23 ± 4 years) ingested 100 g of peanuts together with a lactulose/rhamnose (L/R) solution, followed by rest or by 60 min cycling at 70% of their maximal workload. Significantly higher, though variable, levels of Ara h 6 in serum were found during exercise compared to rest (Peak *p* = 0.03; area under the curve *p* = 0.006), with individual fold changes ranging from no increase to an increase of over 150-fold in the uptake of Ara h 6. Similarly, uptake of lactulose (2–18 fold change, *p* = 0.0009) and L/R ratios (0.4–7.9 fold change, *p* = 0.04) were significantly increased which indicates an increase in intestinal permeability. Intestinal permeability and uptake of Ara h 6 were strongly correlated (*r* = 0.77, *p* < 0.0001 for lactulose and Ara h 6). Endurance exercise after consumption may lead to increased paracellular intestinal uptake of food proteins.

## 1. Introduction

The food and Agriculture Organization of the United Nations predicted that the demand for animal-sourced foods will almost double between 2000 and 2030 due to the fast growth of the global population and changes in consumption patterns with an increase in urbanization in several parts of the world [1]. In order to close the gap between demand and supply, many research initiatives have been taken to study sustainable protein sources for food and feed [2,3,4]. In this quest, there is a strong focus on novel proteins derived from various sources such as plants, algae, food waste, and insects. Next to the technical criteria, questions that arise from the field of novel proteins are directed at their potential effects on human health, with respect to their digestibility and absorption [4], and their potency to activate the immune system [5]. The intestine is of major importance for the proper digestion and absorption of these novel proteins, but it also forms a protective barrier. Changes in intestinal barrier function (e.g., increased permeability) under different physiological conditions may result in increased absorption of large molecules including protein fragments [6,7,8], which could potentially induce an immune response. Mounting evidence indicates that increased intestinal permeability to antigens plays a key role in various inflammatory and autoimmune disorders, underlining the necessity to gain more insight into factors affecting intestinal barrier function [9].

The possibility that the intestine becomes permeable to certain macromolecules, presumably in a variable manner, has been acknowledged for some decades [8,10]. However, only very few studies in humans have investigated the uptake of intact dietary peptides and proteins in a controlled experimental setting [11].

An increased intestinal permeability to immune-reactive proteins or their fragments as a result of intensive exercise has also been postulated as a possible mechanism underlying food-dependent exercise-induced anaphylaxis [12,13].

The aim of the present study was to substantiate the increase in intestinal permeability for dietary proteins upon exercise by determining the effects of a controlled exercise protocol on the uptake kinetics and levels of the peanut allergen Ara h 6 following peanut consumption and to relate this to markers of intestinal permeability and integrity. Ara h 6 was chosen, because, together with Ara h 2, it is the major peanut allergen. Furthermore, Ara h 6 was shown to be resistant to digestion, which increases the possibility of detecting this specific protein in the circulation [14].

## 2. Subjects and Methods

### 2.1. Ethical Approval

This study was approved by the medical ethical committee of Wageningen University (METC-WU 16/11, date of approval: 10 May 2016; Netherlands trial register: NTR5854, PEANUTS Study), and conducted in accordance with the Declaration of Helsinki. All subjects gave their written informed consent after the set-up, objectives and possible consequences of the study had been fully explained.

### 2.2. Subjects

Ten healthy non-atopic subjects, based on exercise-induced effects on intestinal permeability, were recruited via social media and the email database of the university’s Division of Human Nutrition (Wageningen University and Research, Wageningen, The Netherlands). Exclusion criteria were exercise for > three hours per week, smoking, use of drugs, use of nonsteroidal anti-inflammatory drugs (NSAIDs) on a chronic basis, or the use of any medication for gastric or intestinal complaints. Subjects did not perform intense physical activity three days prior to the test days. Subjects also did not eat peanut-containing foods one day nor used alcohol two days prior to the test days. Characteristics of the 10 subjects included in this study are shown in Table 1.

### 2.3. Pre-Study Assessment

Pre-screening was performed prior to subject inclusion to prevent inclusion of subjects in whose serum no Ara h 6 would be reliably detected after peanut intake due to inhibiting effects of the serum matrix; e.g., because of the presence of Ara h 6-specific IgG and particularly IgG4 in the serum, which was found to be common in our study population [14]. This was tested with an in-house developed enzyme-linked immunosorbent assay (ELISA) [14]. Fasted blood samples were tested by spiking 3.1 ng/mL Ara h 6 in 1:1 diluted serum samples. Subjects with a spike recovery <50% were excluded from participation.

A maximal aerobic capacity test (Wmax) was performed using an electronically braked cycle ergometer (Lode Excalibur, Groningen, The Netherlands). After a short warm-up, the subjects started cycling at 100 W. Every minute the power increased with 20 W until they were no longer able to maintain the workload (Wmax; Pedal frequency falling from 90–100 rotations per minute to less than 70).

### 2.4. Study Design

**Figure 1:**
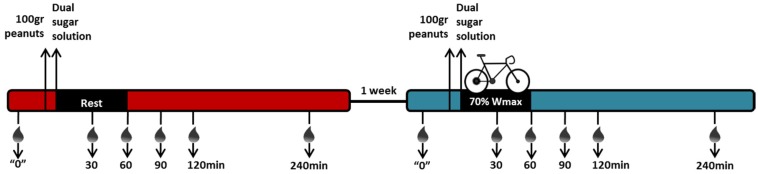
Schematic overview of study set up.

Each subject was tested once in rest and once during and following exercise. Test sessions at the university always started after an overnight fast. Pre-ingestion blood was sampled, after which 100 g of mildly-roasted, unsalted peanuts, obtained from a local supermarket (Albert Heijn; private label product) were consumed within 10 min. This was directly followed by the intake of a solution of two inert, non-digestible, test sugars, containing one gram of l-rhamnose (Carbosynth, Compton, Berkshire, United Kingdom) and 0.5 g of lactulose (Carbosynth) dissolved in 100 mL tap water. Participants were seated comfortably (rest). Blood was sampled in ethylenediaminetetraacetic acid (EDTA) plasma tubes (Vacutainer, Becton Dickinson, Breda, The Netherlands) as well as serum separator tubes (Vacutainer) at an interval of 30, 60, 120, 240, and 360 min after their peanut consumption. EDTA blood tubes were directly centrifuged at 2000× g for 10 min at 4 °C, after which plasma was aliquoted and stored at −80 °C. Serum separator tubes were left to clot for at least 20 min at room temperature, after which the tubes were centrifuged at 2000× g for 10 min at room temperature to obtain serum, which was directly aliquoted and also stored at −80 °C until further analysis. Analysis of Ara h 6 was performed in blood samples collected at *t* = 0, 30, 60, 90, 120, and 240 min. Analysis of fatty acid binding protein-2 (FABP2) was performed in blood samples collected at *t* = 0, 30, and 60 min. Analysis of lactulose and rhamnose was performed in blood samples collected at *t* = 0 and 60 min. One week later, the same procedure was followed, yet this time the intake of the peanuts and the dual sugar solution was followed by endurance exercise. The endurance exercise involved individuals cycling for 60 min at 70% of their Wmax, followed by rest for the remaining three hours. Again, blood was sampled in EDTA plasma tubes as well as serum separator tubes 30, 60, 90 120, and 240 min after finishing their peanut consumption. See Figure 1 for a schematic overview of the intervention and blood sampling.

This scheme shows the blood sampling during rest and exercise. Black box depicts the intervention: 60 min of rest or 60 min of cycling at 70% of the individual’s maximal cycling workload (Wmax). Ara h 6 was analysed at *t* = 0, 30, 60, 90, 120, and 240 min; fatty acid binding protein 2 (FABP2) was analysed at *t* = 0, 30, and 60 min; lactulose and l-rhamnose were analysed at *t* = 0 and 60 min.

### 2.5. Peanut Allergen Ara h 6 Detection in Serum

Serum levels of Ara h 6 were determined with an Ara h 6 ELISA from Indoor Biotechnologies (EL-AH6, Cardiff, UK). The instructions of the manufacturer were followed, except for the reference material, which was replaced by naturally processed Ara h 6, as it occurs next to its native intact form in peanuts [14]. This form of Ara h 6 supposedly better represents Ara h 6 after gastrointestinal digestion [15]. This naturally processed Ara h 6 has been thoroughly tested previously and has shown good reactivity in the assay [14]. The lower level of quantification of Ara h 6 was 0.024 ng/mL. Samples were tested in duplicate and ELISA analyses were performed twice.

### 2.6. Parameters of Intestinal Permeability and Integrity

To assess intestinal permeability, levels of lactulose and l-rhamnose were analyzed in plasma sampled at *t* = 0 and 60 min after intake of the peanuts and the dual sugar solution.

To this end, 125 µL EDTA plasma was transferred to Eppendorf tubes equipped with a 3 kDa cut-off filter (Amicon Ultra 0.5 mL 3 K, Millipore, Etten-Leur, The Netherlands) to remove the plasma proteins. The filter tubes were centrifuged for 30 min at 11,000× g at 4 °C, and clear plasma filtrate was inserted in the pre-cooled sample processor (233 XL, Gilson, Middleton, WI, USA). Sugars were subsequently analysed by LC-MS as described [16]. Plasma lactulose/rhamnose (L/R) ratios were calculated from the 60 min plasma concentrations that were corrected for findings at baseline.

FABP2 was measured as an acute marker of small intestinal epithelial integrity in EDTA plasma (1:1) with an in-house developed ELISA, as described [17].

### 2.7. Statistics

All data is shown as mean ± standard deviation (SD). The Wilcoxon matched-pairs signed rank test was applied to assess changes in area under the curve (AUC), maximal peak height, and time-to-peak analyses of serum levels of Ara h 6 between rest and exercise. Paired *t*-test was used to assess changes in lactulose, rhamnose, and L/R ratio between rest and exercise. Log transformation was applied in case of a deviation from a Gaussian distribution. For rhamnose and L/R ratio analyses, one person was excluded, because no rhamnose could be detected in one of the samples. Two-way analysis of variance with Bonferroni’s multiple comparisons test was used to assess differences in FABP2 levels over time between rest and exercise. Pearson’s correlation coefficients were used to correlate the Ara h 6 parameters, intestinal permeability parameters, and FABP2 levels. All statistics were performed with GraphPad Prism (version 6.07, GraphPad Software Inc., San Diego, CA, USA). Statistical significance was defined as a two-tailed *p* < 0.05.

## 3. Results

### 3.1. Exercise Increases the Levels of Ara h 6 in Serum

**Figure 2:**
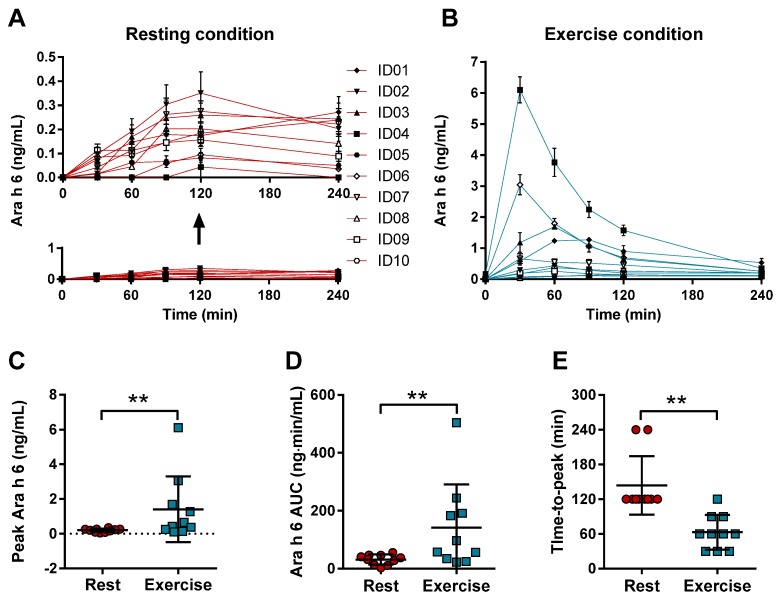
Ara h 6 levels in serum after peanut intake following rest or exercise. Serum levels of Ara h 6 (Mean ± standard deviation) was measured 30, 60, 90, 120, and 240 min after the consumption of 100 g of peanuts. This intake was followed by rest (**A**, **red lines**) or by exercise (60 min of cycling at 70% Wmax) (**B**, **blue lines**). For both conditions, individual peak levels (**C**), area under the curve (AUC) (**D**), and time-to-peak (**E**) were determined for rest (red circle) and exercise (blue square). ID represents an individual * represents a *p*-value < 0.05; ** represents a *p*-value < 0.01.

In rest, consumption of 100 g of peanuts resulted in detectable serum Ara h 6 levels in each of the subjects (Figure 2A). These levels increased when the peanut consumption was followed by exercise, with large inter-individual differences (Figure 2B). One out of the 10 subjects did not show an increase in the peak level of Ara h 6 in exercise compared to rest (Figure 2C), while the other subjects did display an increase. Fold changes ranged from 0.7 to 140.6 and the median peak Ara h 6 increased significantly from 0.22 in rest to 0.55 ng/mL during exercise (*p* = 0.01). The AUC calculated from time point *t* = 0 to *t* = 240 also changed significantly (*p* = 0.006) as the median AUC increased from 34.63 ng·min/mL in rest to 77.10 ng·min/mL in exercise (Figure 2D). Here, individual fold changes ranged from 0.8 to 155.1 fold. Compared to rest, the peak concentration of Ara h 6 in serum after peanut intake was reached earlier in time (*p* = 0.004) during exercise (Figure 2E).

### 3.2. Exercise-Induced Effects on Intestinal Permeability and Integrity Markers

**Figure 3:**
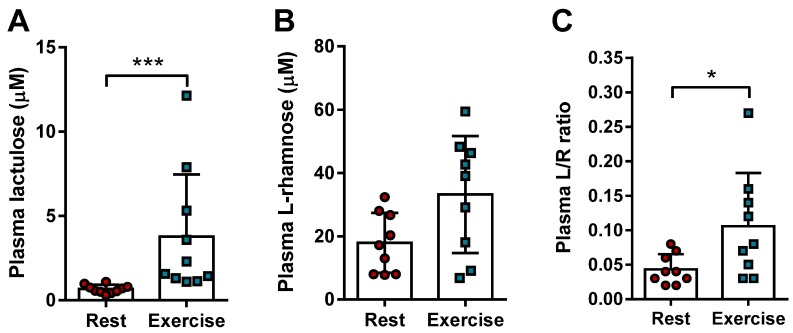
Lactulose and l-rhamnose sugar test for the analysis of intestinal permeability during rest or exercise. Plasma levels of lactulose (**A**) and l-rhamnose (**B**) (Mean ± SD) were measured after intake of the dual sugar solution followed by rest (red circle) or exercise (blue square). From these, the Lactulose-over-l-rhamnose (L/R) ratio was calculated (**C**). * represents a *p*-value < 0.05; *** represents a *p*-value < 0.001.

Exercise-induced changes in integrity of the small intestine can be experimentally assessed by its permeability to small compounds, such as the inert sugar lactulose [18,19,20,21]; and by circulating markers of intestinal integrity, such as FABP2 [22]. Plasma levels of lactulose increased significantly (*p* = 0.0009) when consumption of peanuts is followed by exercise compared to rest (Figure 3A). There was no difference in the absorption of l-rhamnose (Figure 3B), although one could see a tendency towards increased absorption (*p* = 0.08). Nevertheless, the calculated L/R ratios showed a significant increase (*p* = 0.04) as individual ratio fold changes ranged from 0.4 to 7.9-fold. These results suggest an increase in paracellular intestinal permeability when consumption of peanuts is followed by exercise.

**Figure 4:**
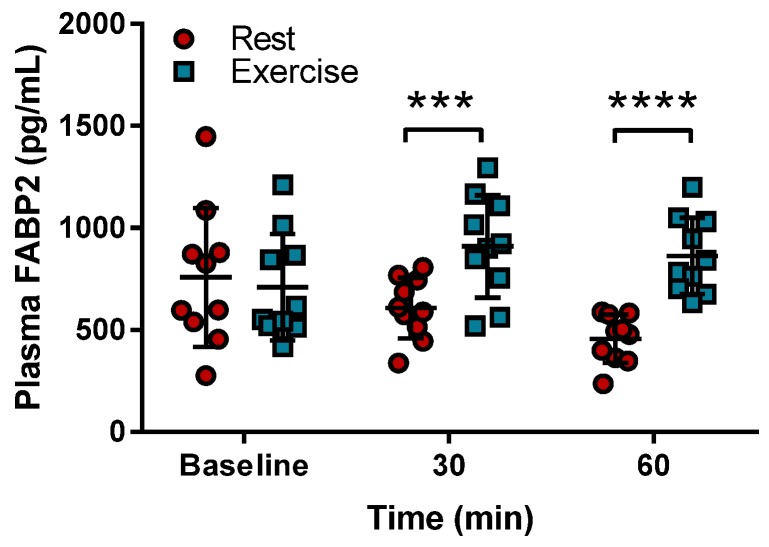
Circulating fatty acid binding protein-2 (FABP2) levels upon exercise. Fatty acid binding protein-2 levels (Mean ± standard deviation) were measured in plasma as a marker of intestinal integrity after 30 min or 60 min during rest (red circles) or exercise (blue squares). *** represents a *p*-value < 0.001; **** represents a *p*-value < 0.0001.

The effect of peanut consumption and endurance exercise on intestinal integrity was determined with circulating levels of FABP2. Figure 4 shows that plasma FABP2 levels were increased significantly after 30 min of exercise compared to the levels when the peanuts were consumed followed by rest. This increase became more pronounced after 60 min of exercise when compared to their values during rest.

### 3.3. Correlations of Ara h 6 Levels and Intestinal Permeability and Integrity Markers

**Figure 5:**
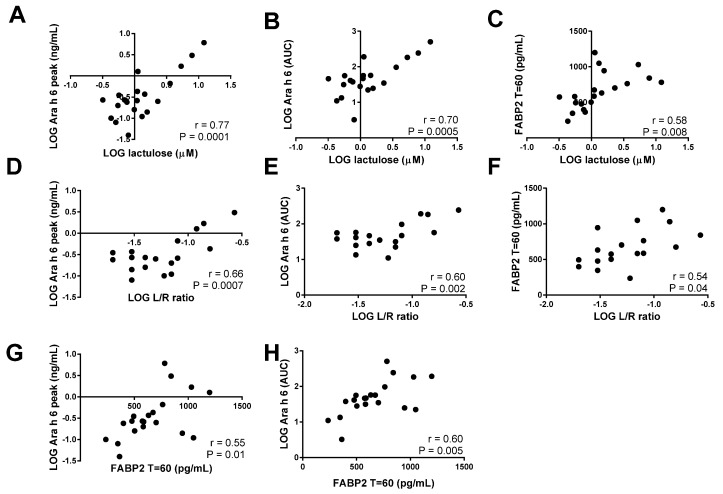
Correlation analysis of intestinal integrity markers and levels of Ara h 6. Correlations are shown with the calculated Pearson’s correlation coefficients (*r*) and statistical significance. Correlations are made between the lactulose levels and the peak levels of Ara h 6 (**A**) and the Ara h 6 AUC values (**B**), between the Lactulose-over-l-rhamnose (L/R) ratio and the peak levels of Ara h 6 (**D**) and the Ara h 6 area under the curve (AUC) values (**E**), and between fatty acid binding protein 2 (FABP2) levels after 60 min and lactulose levels (**C**), L/R ratio (**F**), Ara h 6 peak levels (**G**), and Ara h 6 AUC (**H**). LOG = logarithm.

Pearson correlation coefficients were calculated to determine the correlation between the uptake of Ara h 6 and intestinal permeability as reflected by the uptake of lactulose. There was a strong correlation between the lactulose levels both with the peak levels of Ara h 6 (*r* = 0.77; *p* < 0.0001; Figure 5A) and with the Ara h 6 AUC values (*r* = 0.70; *p* = 0.0005; Figure 5B). The L/R ratio also correlated well with the peak levels of Ara h 6 (*r* = 0.66; *p* = 0.0007; Figure 5D) and the Ara h 6 AUC values (*r* = 0.60; *p* = 0.002; Figure 5E). The correlations between FABP2 levels after 60 min and lactulose levels (*r* = 0.58, *p* = 0.008; Figure 5C), L/R ratio (*r* = 0.54, *p* = 0.04; Figure 5F), Ara h 6 peak levels (*r* = 0.55, *p* = 0.01; Figure 5G), and Ara h 6 AUC (*r* = 0.60, *p* = 0.005; Figure 5H) were slightly lower, but still significant.

## 4. Discussion

The aim of the present study was to determine the effects of endurance exercise on intestinal permeability to the peanut-derived allergen Ara h 6 and to compare this with commonly used small molecule markers for intestinal permeability. Although exercise-induced permeability towards potential allergens has been hypothesized to play a role in food-dependent exercise-induced anaphylaxis, the experimental evidence in humans is limited so far [23,24].

The current study shows a clear increase in the level of Ara h 6 as measured by ELISA in serum when peanut consumption was followed by exercise compared to the resting condition. Cycling at 70% Wmax also resulted in increased lactulose levels and consequently increased L/R ratios compared to rest. This outcome supports the hypothesis that exercise leads to increased paracellular permeability of the small intestine towards small molecules [25]. The present study furthermore indicates a high correlation between the levels of Ara h 6 and those of lactulose. It is therefore enticing to suggest a direct role for increased intestinal paracellular permeability in absorption of this allergen. There is a rather high variation in effect between the individuals and these differences appear to be similar for the effect on the levels of Ara h 6 as well as the L/R ratio. The causes for such inter-individual differences are not known.

Although assumptions are often made, there is limited evidence besides our study for the passage of larger food-derived proteins and peptides across the intestinal barrier into the circulation [6,26,27,28,29,30], in particular in combination with exercise-induced permeability. Matsuo et al. [12] showed increased levels of circulating gliadin peptides upon exercise and intake of NSAIDs, but did not assess intestinal permeability. In recent studies, we used a strenuous exercise protocol involving both a glycogen depletion interval phase and an endurance exercise phase, which when compared to resting conditions, resulted in an increase in intestinal permeability to lactulose [31,32]. In the same model we detected an exercise-induced increase of the urinary recovery of the small casein-derived peptide betacasomorphin-7 [32]. However, the question for the uptake of larger dietary protein in the blood in combination with exercise remained. It has been demonstrated that the peanut protein Ara h 2, which together with Ara h 6, are the most potent peanut allergens [33,34], can be detected in breast milk after peanut ingestion [35], but detection in blood has not yet been proven and the effect of exercise not studied. Although peanut consumption and Ara h 6 detection are included in the resting condition, performing an exercise only intervention could additionally provide information on a possible interaction between peanut consumption and exercise on intestinal permeability.

Because little is known on the absorption and clearance mechanisms of Ara h 6, or its derived peptides, in principle the increase in serum Ara h 6 peak levels could result from increased passage across the intestinal epithelial barrier, from lower clearance rate, or a combination of the two. However, given the effects seen on markers for intestinal permeability, and the strong correlation between those parameters, an increased passage seems conceivable. Theoretically, changes in intestinal digestion due to exercise could lead to changes in the detection of Ara h 6. It seems likely, however, that the allergen is crossing the barrier relatively intact. Digestion of Ara h 6 by pepsin and trypsin has been investigated in the past, showing that these only partially hydrolyse Ara h 6, resulting in two large peptides of around 4 to 5 kDa and 10 kDa in size, which are bound to each other by disulphide bridges [15,36,37]. We cannot claim with certainty whether it is the intact Ara h 6 protein or these two bridged peptides that are appearing in the serum. These peptides are comparably reactive with intact Ara h 6 in the ELISA method that has been used [14]. The uptake of Ara h 6 and its immunoreactive fragments that are detected are physiologically relevant as these fragments are thought to contribute to the allergenicity of peanuts. Ara h 1 and Ara h 3 are much more sensitive to digestion compared to Ara h 6 and Ara h 2, making them less able to trigger an immune response [36,38,39]. This study reports for the first time the detection of Ara h 6 in the blood. Although physiological levels of Ara h 6 are not exactly known, the levels found in the current study is comparable to the levels of Ara h 2 in the circulation after peanut intake found by others [30,38].

In most individuals, the highest Ara h 6 level in serum was found 120 min after peanut intake, which is similar in time as found in our prior study [14]. This peak, however, clearly shifted to earlier time points when the intake of peanuts was followed by intense exercise. This is possibly due to an increased gastric emptying rate of a solid meal during exercise [40]. Alternatively, this may be related to a forward shift in steady state [41] or changes in the route of epithelial transport.

In the current study, a median peak level of 0.55 ng/mL during exercise reflects about 0.0002% of the ingested dose of Ara h 6. This level lies within the suggested scope of antigen sampling, which takes place via transcytosis and sampling by dendritic cells. This process is estimated to occur at rates which are several orders of magnitude smaller than 0.1% of the administered dose [10,42]. These values, however, are mostly based on animal models or in vitro testing and only limited data in humans is available. The occurrence of food-dependent exercise-dependent anaphylaxis suggests that increased absorption during exercise is clinically relevant, as the threshold for allergic symptoms could be reached more easily at a similar intake.

## 5. Conclusions

We show that endurance exercise after peanut consumption results in a clear increase in the serum levels of Ara h 6 after peanut consumption, which correlates strongly with increased intestinal permeability. This suggests that exercise has a profound effect on the amount of food-derived allergen crossing the epithelial barrier, also in healthy non-sensitized individuals. This effect is possibly mediated by an increase in intestinal permeability. The current model could be used to screen for the potential health effects of novel protein sources under different physiological conditions.

## Figures and Tables

**Table 1:** Subject characteristics.

ID	M/F	Age (Years)	BMI (kg/m^2^)	Wmax (watt)	Consumption of Products with High Peanut Content ^1^	Consumption of Products with Low Peanut Content ^2^
1	F	22	24.9	240	1 × per week	<1 × per month
2	F	21	22.7	200	<1 × per month	1–3 × per month
3	F	22	25.6	320	1–3 × per month	1–3 × per month
4	F	22	25.2	280	(Almost) daily	1–3 × per month
5	M	20	24.8	320	1 × per week	1–3 × per month
6	M	21	24.7	360	1 × per week	1 × per week
7	M	26	22.7	300	1–3 × per month	<1 × per month
8	M	32	23.5	300	1 × per week	<1 × per month
9	F	22	22.8	260	(Almost) daily	<1 × per month
10	F	23	23.6	200	(Almost) daily	1–3 × per month

^1^ Examples: peanuts/peanut butter/peanut sauce; ^2^ Examples: candy bars/cookies/muesli (bars). Wmax = Maximal cycling workload. M/F = Male/Female, BMI = Body Mass Index.
